# Genome-Wide Identification of DNA Binding with One Finger (*Dof*) Gene Family in Tartary Buckwheat (*Fagopyrum tataricum*) and Analysis of Its Expression Pattern after Exogenous Hormone Stimulation

**DOI:** 10.3390/biology11020173

**Published:** 2022-01-21

**Authors:** Jing Li, Yuchuan Zhang, Lei Xu, Chenyang Wang, Yan Luo, Shan Feng, Yuhao Yuan, Qinghua Yang, Baili Feng

**Affiliations:** 1 State Key Laboratory of Crop Stress Biology for Arid Areas, College of Agronomy, Northwest A&F University, Xianyang 712000, China; lijing1993@nwafu.edu.cn (J.L.); zhangyuchuan@nwafu.edu.cn (Y.Z.); 2020055040@nwafu.edu.cn (L.X.); wcy410223@163.com (C.W.); 1994lyyl@nwsuaf.edu.cn (Y.L.); yuanyuhao@nwafu.edu.cn (Y.Y.); 2016060037@nwsuaf.edu.cn (Q.Y.); 2 School of Mathematics and Statistics, Northwestern Polytechnical University, Xi’an 710129, China; fengshan1912@mail.nwpu.edu.cn

**Keywords:** Tartary buckwheat, *Dof*, motif, *cis*-acting elements, synteny analysis, hormone

## Abstract

**Simple Summary:**

A number of studies have demonstrated that DNA binding with one finger (*Dof*) proteins are involved in multiple biological processes. In the present study, *Dof* genes or proteins in Tartary buckwheat (*FtDofs*) were systematically analysed, including their physical properties, phylogenetic relationships, structure, motif composition, *cis*-acting elements present in promoter regions, chromosomal distribution, gene duplication events, syntenic relationships, expression patterns in different tissues and different fruit developmental stages and responses to exogenous hormone stimulation. The results indicated that the expansion of *FtDofs* was mainly due to segmental duplication. The tissue-specific expression patterns of *FtDofs* and their positive responses to exogenous hormone stimulation suggest that they play important roles in the growth and development of Tartary buckwheat as well as in the adaptation to environmental changes. Collectively, this study lays a foundation for further exploration of the function of *FtDof* genes in Tartary buckwheat.

**Abstract:**

DNA binding with one finger (*Dof*) proteins have been proven to be involved in multiple biological processes. However, genome-wide identification of the *Dof* gene family has not been reported for Tartary buckwheat (*Fagopyrum tataricum*). In this study, 35 *Ft*Dof proteins were identified, and they could be divided into nine phylogenetic subgroups. Proteins within the same subgroup had similar gene structure and motif composition. Moreover, abundant *cis*-acting elements were present in the promoter regions of *FtDof* genes. Segmental duplication was the primary driving force for the evolution of the *Ft*Dof gene family. Synteny analysis indicated that Tartary buckwheat was closer to dicotyledons, and more orthologous *Dof* genes existed among them. The expression pattern of *FtDofs* in different tissues and at different fruit developmental stages varied. Different tissues contained several genes that were specifically expressed. *FtDof* expression was mainly upregulated under methyl jasmonate treatment and downregulated under other hormone treatments. Taken together, *FtDofs* may play important roles in the growth and development of Tartary buckwheat and in response to abiotic and biotic stresses. Therefore, the genome-wide identification and expression pattern analysis of the Tartary buckwheat *Dof* gene family lays a foundation for further exploration of the functional characteristics of *FtDofs* in the future.

## 1. Introduction

Transcription factors (TFs) participate in recruiting and recognizing specific DNA sequence elements in the promoter region of genes to regulate the spatiotemporal expression of target genes and further control or influence several biological processes [1]. DNA binding with one finger (*Dof*) proteins are a subfamily of the zinc finger protein family; they are ubiquitous but plant-specific (they have not been found in other eukaryotes, such as yeast or animals) [2,3]. *Dof* proteins are usually made up of 200–400 amino acids, and they have a highly conserved *Dof* domain composed of 50–52 amino acids at the N-terminus [2], a nuclear localisation signal and a transcriptional regulatory domain at the C-terminus, which varies greatly [4]. The *Dof* domain has a Cys2/Cys2 zinc finger structure, which can bind to the AAAG *cis*-element in the promoter region of the target gene and is considered as a bifunctional domain that plays key roles in DNA-binding and protein–protein interaction activities [2,5,6].

The first *Dof* protein was cloned from maize leaves (*ZmDof1*) and preliminarily considered as a DNA-binding protein [7]. *Dof* genes have recently been identified in numerous plants with the completion of genome sequencing of plant species. The number of *Dof* genes greatly varies among different species; for example, there are 36 in *Arabidopsis thaliana* [8], 30 in rice (*Oryza sativa*) [8], 26 in barley (*Hordeum vulgare*) [9], 46 in maize (*Zea mays*) [10], 28 in sorghum (*Sorghum bicolor*) [11], 34 in tomato (*Solanum lycopersicum*) [12] and 96 in wheat (*Triticum aestivum*) [13]. Furthermore, a number of studies have evidenced the unique role of *Dof* proteins in multiple biological processes, including tissue differentiation, seed development and regulation of metabolism [14], vascular tissue development [15], stomatal maturation and functioning [16], flowering time [17,18], pollen maturation [19], seed germination [20,21], endosperm development [22], carbon and nitrogen metabolism [23] and responses to phytohormones [24,25,26,27], as well as biotic and abiotic stresses [28,29,30]. These findings proved that *Dof* proteins play crucial roles in the growth and development of plants, and these TFs also play a role in the response to abiotic and biotic stresses. However, to the best of our knowledge, genome-wide identification and relevant studies of the *Dof* gene family have not been reported for Tartary buckwheat.

Tartary buckwheat (*Fagopyrum tataricum*) is an edible and medicinal crop originating from China. The grains of Tartary buckwheat are gluten-free and rich in bioflavonoids, which have many important biological functions; therefore, the Tartary buckwheat grain has been praised as one of the 21st century’s green foods [31,32]. In our previous research, we found that the leaves and flowers of Tartary buckwheat are also rich in flavonoids, indicating its potential application in pharmaceutical and food industries [33,34]. However, the weak research foundation for Tartary buckwheat makes it difficult to meet the growing demand for in-depth development and utilisation [35]. A high-quality, chromosome-scale Tartary buckwheat genome was recently reported [36], thus making it possible to systematically study some gene families with important functions on the basis of the whole genome. In the present study, the characteristics, evolution and expression of *Dof* genes in Tartary buckwheat were comprehensively analysed to lay a foundation for subsequent studies on the function of *FtDof* genes.

## 2. Materials and Methods

### 2.1. Identification of Dof Family Genes in Tartary buckwheat

A total of 36 *Dof* proteins of *A. thaliana* obtained from the TAIR database (https://www.arabidopsis.org/, accessed on 1 September 2020) were used for BLASTP searches to retrieve possible *Ft*Dofs in the Tartary buckwheat genome (http://www.mbkbase.org/Pinku1/, accessed on 1 September 2020), with e-value ≤ 1 × 10^−10^ and score value ≥ 100, to identify the *Dof* family genes in Tartary buckwheat [37]. Then, the hidden Markov model profile of the *Dof* domain (PF02701) acquired from the Pfam database (http://pfam.xfam.org/, accessed on 1 September 2020) was used to retrieve *Ft*Dofs from the Tartary buckwheat genome by using HMMER3.3 with default parameters. Furthermore, the putative sequences were submitted to the Conserved Domain Database (CDD, https://www.ncbi.nlm.nih.gov/cdd, accessed on 13 October 2020), SMART (http://smart.embl-heidelberg.de/, accessed on 2 September 2020) and Pfam (accessed on 15 October 2020) for verifying the existence of the *Dof* core sequences. Subcellular localisation was predicted by CELLO (http://cello.life.nctu.edu.tw/, accessed on 5 September 2020). Protein molecular weight (Mw) and theoretical isoelectric point (*p*I) were computed via Expasy (https://web.expasy.org/compute_pi/, accessed on 5 September 2020).

### 2.2. Phylogenetic Analyses and Classification of FtDof Family Members

The phylogenetic tree of the *Dof* proteins from *A. thaliana* and Tartary buckwheat was constructed using the maximum likelihood (ML) method with MEGA X (version 10.0.4, Mega Limited, Auckland, New Zealand) [38]. The protein sequences of *Ft*Dofs and *At*Dofs were first aligned using MUSCLE with default settings [39]. Then, the best model of the ML method was calculated using MEGA X. Finally, the JTT + G + I + F model was chosen to construct the phylogenetic tree. The bootstrap value was set to 1000, and all positions with less than 80% site coverage were eliminated. Then, the *Ft*Dofs were grouped in accordance with the classification of *At*Dofs [8].

### 2.3. Sequence Characteristic Analysis

The exon–intron structures of *FtDofs* and the presence or absence of the *Dof* domain were analysed on the basis of CDD results by using TBtools (version v1.0986853, https://github.com/CJ-Chen/TBtools, accessed on 5 September 2020) [40] to investigate the differences in genes and proteins among the *Ft*Dofs family. The conserved motifs of the *Dof* proteins from Tartary buckwheat were searched using the MEME Suite (https://meme-suite.org/meme/tools/meme, accessed on 5 September 2020), where the maximum number of motifs was set to 10 and the remaining parameters were set to default values. The *cis*-acting elements in the 2.0 kb promoter region upstream of the *FtDofs* were analysed using PlantCARE (http://bioinformatics.psb.ugent.be/webtools/plantcare/html/, accessed on 9 September 2020).

### 2.4. Chromosome Location, Gene Duplication and Synteny Analysis

Detailed information on the identified *FtDofs* was obtained from the Tartary buckwheat genome project, and then the genes were numbered in accordance with their distribution on chromosomes. The multiple collinear scanning toolkit (MCScanX, http://chibba.pgml.uga.edu/mcscan2/, accessed on 6 September 2020) was used to detect gene duplication events [41]. Syntenic analyses were conducted on *Ft*Dofs and the *Dof* family protein sequences of *Glycine max*, *S. lycopersicum*, *Vitis vinifera*, *O. sativa*, *Setaria italica* and *S. bicolor* obtained from PlantTFDB v5.0 (http://planttfdb.gao-lab.org/, accessed on 10 September 2020) together with *At*Dofs obtained from the TAIR database by using TBtools.

### 2.5. Plant Materials and Treatments

The Tartary buckwheat accession ‘Zhenba-3’ used in this study was provided by the Minor Grain Research Centre, Northwest A & F University, Yangling, Shaanxi, China. The variety was cultivated at the test site of the Baoji Academy of Agricultural Sciences, Shaanxi, China, on 25 June 2019. The region of the test site has a warm temperate semi-humid climate. The management measures during the growth of Tartary buckwheat were implemented in accordance with the local production practice and crop demand. The roots, stems and leaves of Tartary buckwheat were collected at the squaring stage, and the fruits were collected at four different developmental stages, i.e., 3, 10, 17 and 24 days after pollination, corresponding to the initial formation stage (F_S1), green fruit stage (F_S2), discolouration stage (F_S3) and initial maturity stage (F_S4), respectively. All samples were collected from at least three plants and then immediately frozen in liquid nitrogen.

Different exogenous hormone treatments were conducted to investigate the Tartary buckwheat *Dof* gene family’s response to hormones. The Tartary buckwheat accession ‘Zhenba-3’ was planted in the experimental site of Northwest A & F University. Furthermore, when seedlings grew to the three-leaf one-heart stage, they were treated with 100 μM methyl jasmonate (MeJA), 100 μM abscisic acid (ABA), 100 μM salicylic acid (SA), 10 μM indole-3-acetic acid (IAA) and 10 μM gibberellin (GA) by foliar spray and treated with deionised water as a blank control. The second leaf of the seedling was harvested 6 h after treatments [42] and then quickly frozen in liquid nitrogen. Three biological replicates were performed for each sample.

### 2.6. Expression Analyses of FtDof Genes by qRT-PCR

Total RNA was extracted with Plant RNA Extraction Kit (TaKaRa, Takara Bio Inc., Tokyo, Japan) and treated with DNase I (TaKaRa, Takara Bio Inc., Tokyo, Japan) to remove genomic DNA. Then, approximately 2 μg of the total RNA was used to synthesise cDNA with PrimeScript™ II 1st Strand cDNA Synthesis Kit (TaKaRa, Takara Bio Inc., Tokyo, Japan) in accordance with the manufacturer’s instructions. The coding sequences (CDSs) of *FtDofs* were obtained from the Tartary buckwheat genome project. Primers for qRT-PCR (Table S1) were designed with Primer3 (version 4.1.0, https://primer3.ut.ee/, accessed on 10 June 2021), and the amplification efficiency was more than 90%. qRT-PCR was performed on the Q7 Real-Time PCR System (Applied Biosystems™, Foster City, CA, USA) using the TB Green™ *Premix Ex Taq*™ II (TaKaRa, Takara Bio Inc., Tokyo, Japan). The Tartary buckwheat histone H3-encoding gene was selected as the internal reference gene, and calculation was carried out in accordance with the 2^−(ΔΔCt)^ method [43]. The expression data of each gene were first normalised by the Z-score method and then drawn as a heatmap. Each treatment included three biological replicates and three technical replicates. Finally, Student’s *t*-test was conducted using R software to examine whether the expression of *FtDofs* changed significantly after exogenous hormone treatments compared with that after control treatment.

### 2.7. Gene Ontology (GO) and Kyoto Encyclopedia of Genes and Genomes (KEGG) Enrichment Analysis of FtDof Proteins

GO and KEGG enrichment analyses were conducted using online OmicShare tools (https://www.omicshare.com/tools, accessed on 28 August 2021).

## 3. Results

### 3.1. Genome-Wide Identification of FtDofs in Tartary Buckwheat

**Figure 1:**
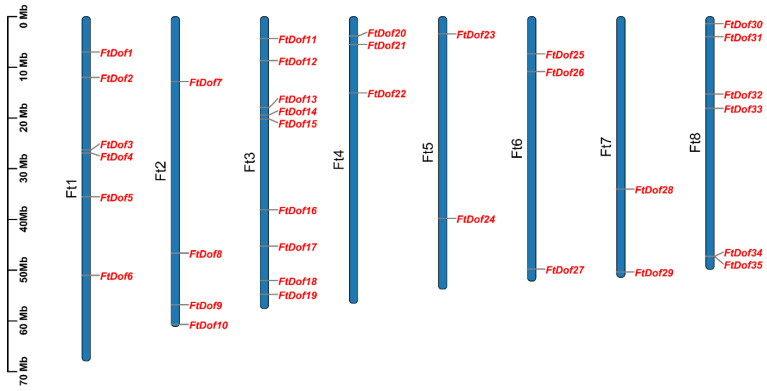
Schematic of chromosomal distribution of Tartary buckwheat *Dof* genes.

Through retrieval and subsequent multi-verification, 35 *Dof* proteinswere identified in the Tartary buckwheat genome (Table S2). In accordance with the distribution order of *Dof* genes on the chromosome, *FtDofs* were assigned names from *FtDof1* to *FtDof35* (Figure 1). Then, the basic characteristics of *FtDofs*, including CDS length, protein length, Mw, *p*I and subcellular localisation, were investigated (Table 1). The CDS length of *FtDofs* ranged from 393 bp (*FtDof28*) to 1416 bp (*FtDof25*), the amino acid length of the corresponding proteins varied from 130 aa to 471 aa, and their Mw ranged from 14.8 kDa to 50.8 kDa. The *p*I of *Ft*Dof proteins ranged from 4.46 (*FtDof8*) to 9.84 (*FtDof7*). The results of subcellular localisation prediction indicated that all *Ft*Dof proteins are localised in the nucleus.

### 3.2. Phylogenetic Analysis and Classification of FtDof Proteins

**Figure 2:**
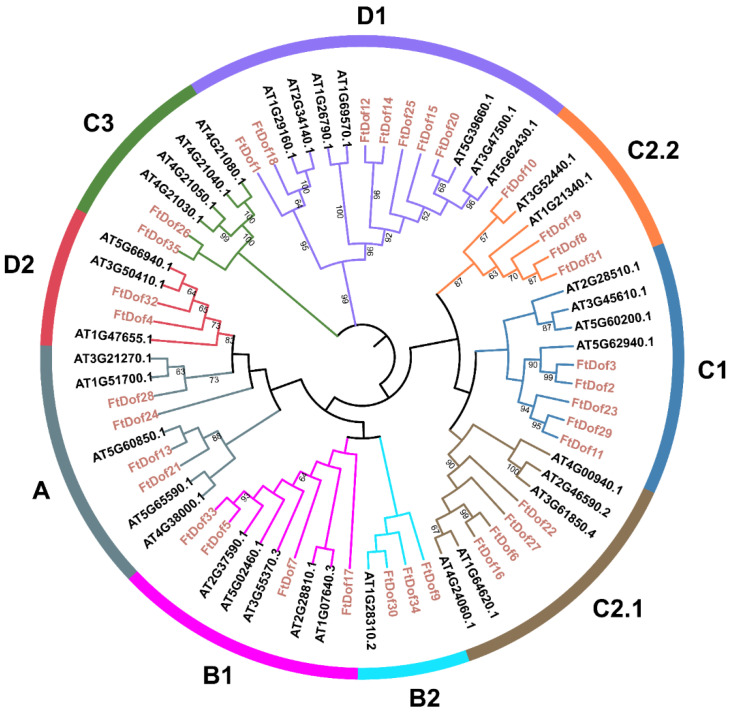
Phylogenetic tree constructed with *Dof* proteins from Tartary buckwheat and *Arabidopsis thaliana* by MEGA X (version 10.0.4) based on maximum likelihood method.

A phylogenetic tree was constructed on the basis of 35 *Ft*Dofs identified in this study, together with 36 *Dof* proteins from *A. thaliana*, to explore the evolutionary relationships of *Dof* proteins in Tartary buckwheat (Figure 2). The result showed that 71 *Dof* proteins were classified into four major groups (A, B, C and D) and further divided into nine subgroups (A, B1, B2, C1, C2.1, C2.2, C3, D1 and D2). Each group or subgroup contained *Ft*Dofs, but their distributions were heterogeneous. Group C was the largest group, containing 15 genes and accounting for 42.86% of the total number of *Ft*Dofs, whereas group A was the smallest group and contained only 4 genes, accounting for 11.43% of the total number of *Ft*Dofs. Groups B and D contained seven and nine *Ft*Dofs, respectively.

### 3.3. Gene Structure and Conserved Motif Analysis of FtDof Genes

**Figure 3:**
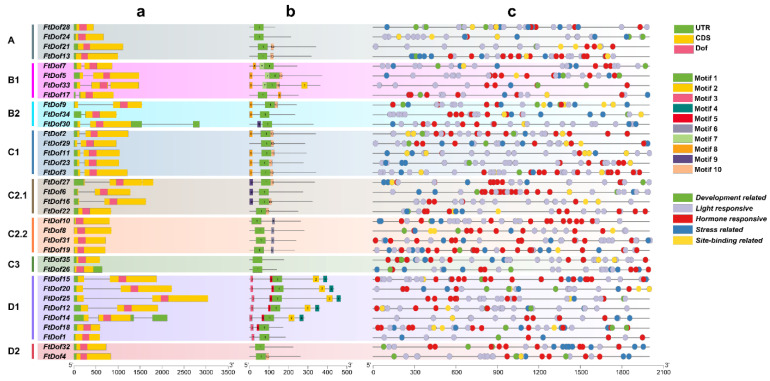
Gene structure, motif composition and *cis*-acting element distribution of *Dof* genes in Tartary buckwheat. (**a**) Exon–intron structures of *Dof* genes in Tartary buckwheat. The green box represents the untranslated region, the yellow box represents the coding sequence, and the red box represents the *Dof* domain. (**b**) Motif composition of *Dof* proteins in Tartary buckwheat. As shown in the legend on the right, Motifs 1–10 are marked with different colours. (**c**) Distribution of *cis*-acting elements in the promoter region of Tartary buckwheat *Dof* genes. Different types of *cis*-elements are marked with different colours, as indicated in the legend to the right.

The exon–intron structure of the 35 *FtDofs* was analysed on the basis of genomic DNA sequences (Figure 3a). Generally, the difference in the number of introns in each *FtDof* gene was very small, ranging from 0 to 2. Only three *FtDof* genes (*FtDof33*, *FtDof30* and *FtDof14*) contained two introns, and they belonged to subgroups B1, B2 and D1, respectively. A total of 15 *FtDof* gene members had no introns (42.86%), and 17 members had only one intron (48.57%). Interestingly, the *FtDof* gene members belonging to the C1 subgroup contained only one intron, and the genes belonging to the A, C2.2, C3 and D2 subgroups lacked introns, indicating that the *FtDof* genes from the same subgroup have a similar gene structure.

The putative motifs were analysed by MEME to investigate the diversity of motif compositions among different *Ft*Dof proteins. As shown in Figure 3b, 10 distinct motifs were identified (Table S3). Motif 1 was present in all *Ft*Dofs, which corresponded to the conserved *Dof* domain. Moreover, motif 10 was widely present in 14 *Ft*Dof proteins. Therefore, it was considered to be another conserved domain. *Ft*Dof proteins belonging to the same subgroup had similar motif composition. Motif 2 was mainly present in the D1 subgroup and one member of the B1 subgroup (*Ft*Dof33). Motifs 3, 4 and 5 only appeared in the D1 subgroup, where all *Ft*Dof proteins in the D1 subgroup contained motif 3. Motifs 6 and 7 were exclusively present in subgroups C2.2 and B1, respectively. Motif 8 was widely present but limited to B1, B2 and C1 subgroups. Motif 9 was present in the C2.1 subgroup except for *Ft*Dof22 and one member of the B2 subgroup (*Ft*Dof30). In general, similar to gene structure, *Ft*Dof proteins belonging to the same subgroup possessed similar motif composition.

### 3.4. Promoter cis-Acting Element Analysis of FtDof Genes

The *cis*-acting elements in the promoter regions were investigated to predict the potential functions of the *FtDofs* (Figure 3c and Table S4). Light-responsive elements were widely present in all *FtDof* genes. Furthermore, some development-related elements (e.g., AACA_motif, GCN4_motif and CAT-box), stress-related elements (e.g., LTR, MBS and WUN-motif) and site-binding-related elements (e.g., MBSI, CCAAT-box and AT-rich element) were still present in the *FtDof* genes, but not every *FtDof* gene contained them. Notably, almost all *FtDof* genes had a considerable number of hormone-responsive elements (e.g., GARE-motif, P-box and TATC-box), though the specific number differed, ranging from 3 to 24, indicating that the response of different *FtDof* genes to hormones may vary greatly. Interestingly, the *cis*-acting elements involved in ABA responsiveness (W-box and ABRE) and MeJA responsiveness (CGTCA-motif and TGACG-motif) were the most abundant among the hormone-responsive elements, reaching 191 and 100, respectively. The results suggest that *FtDof* genes may play an important role in hormone signal transduction, especially in response to ABA and MeJA.

### 3.5. Gene Duplication and Synteny Analysis of FtDof Proteins

**Figure 4:**
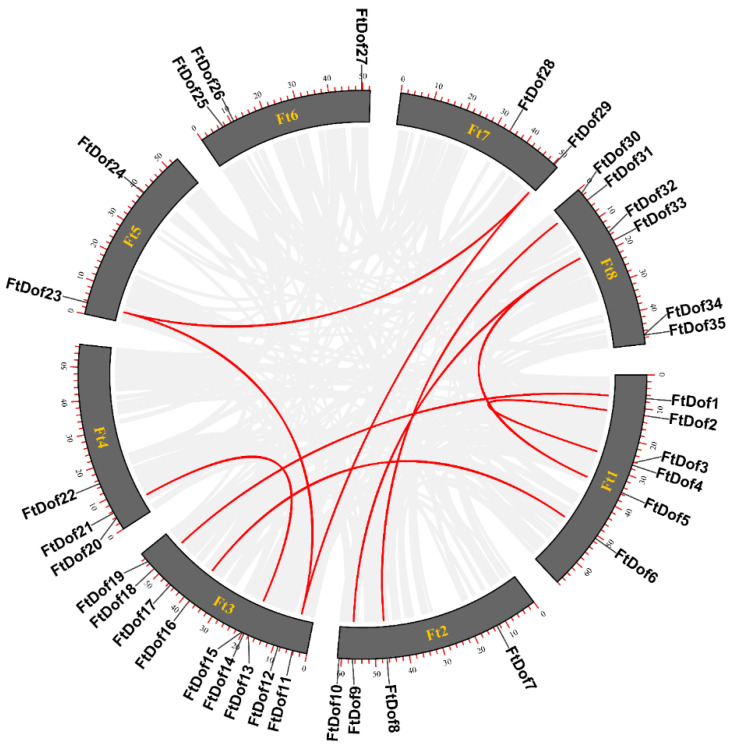
Schematic of interchromosomal relationship of Tartary buckwheat *Dof* proteins. Red lines represent segmental duplication events.

As shown in Figure 1, 35 *FtDof* genes were unevenly distributed on the eight chromosomes of Tartary buckwheat, with six, four, nine, three, two, three, two and six genes, respectively. The MCScanX program was used to investigate whether gene duplication events took place in the Tartary buckwheat genome. The results showed no tandem duplication events amongst the *Ft*Dofs. However, 10 pairs of segmental duplication events were found, namely, *Ft*Dof1/*Ft*Dof18, *Ft*Dof2/*Ft*Dof3, *Ft*Dof5/*Ft*Dof33, *Ft*Dof6/*Ft*Dof16, *Ft*Dof8/*Ft*Dof31, *Ft*Dof9/*Ft*Dof33, *Ft*Dof11/*Ft*Dof23, *Ft*Dof11/*Ft*Dof29, *Ft*Dof13/*Ft*Dof21 and *Ft*Dof23/*Ft*Dof29 (Figure 4), indicating that segmental duplication was the primary driving force for the evolution of the *Ft*Dof gene family.

**Figure 5:**
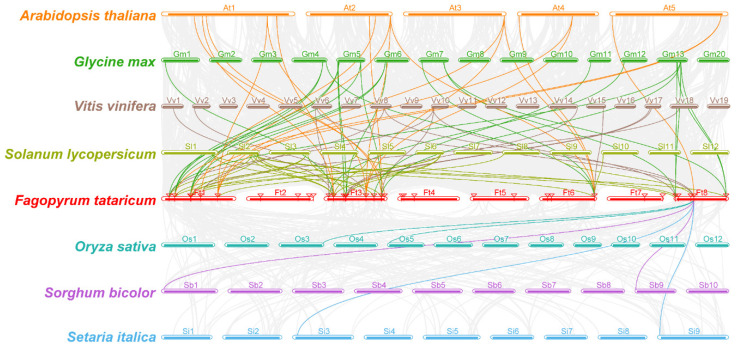
Synteny analysis between the *Dof* proteins of Tartary buckwheat with four dicotyledonous plants (*Arabidopsis thaliana*, *Solanum lycopersicum*, *Glycine max* and *Vitis vinifera*) and three monocotyledonous plants (*Oryza sativa*, *Setaria italica* and *Sorghum bicolor*) by MCScanX.

A synteny analysis on the *Dof* proteins from Tartary buckwheat and four dicotyledonous crops (*A. thaliana*, *S. lycopersicum*, *G. max* and *V. vinifera*) and three monocotyledonous crops (*O. sativa*, *S. bicolor* and *S. italica*) was conducted with MCScanX to explore the evolutionary relationship between *Dof* proteins from Tartary buckwheat and other representative crops (Figure 5). The numbers of gene pairs homologous to *Ft*Dofs were 20 (*A. thaliana*), 34 (*G. max*), 14 (*V. vinifera*), 24 (*S. lycopersicum*), 2 (*O. sativa*), 2 (*S. bicolor*) and 2 (*S. italica*) (Table S5). Generally, Tartary buckwheat had more orthologous genes with dicotyledons than with monocotyledons. In particular, *Ft*Dofs had the most collinear gene pairs within *G. max*, which were *Ft*Dof2, *Ft*Dof3, *Ft*Dof30 and *Ft*Dof34. Interestingly, *Ft*Dof33 had collinear gene pairs with plants other than *G. max* and two collinear gene pairs within all monocotyledons.

### 3.6. Expression Pattern of FtDof Genes in Different Tissues and in Different Fruit Developmental Stages of Tartary Buckwheat

**Figure 6:**
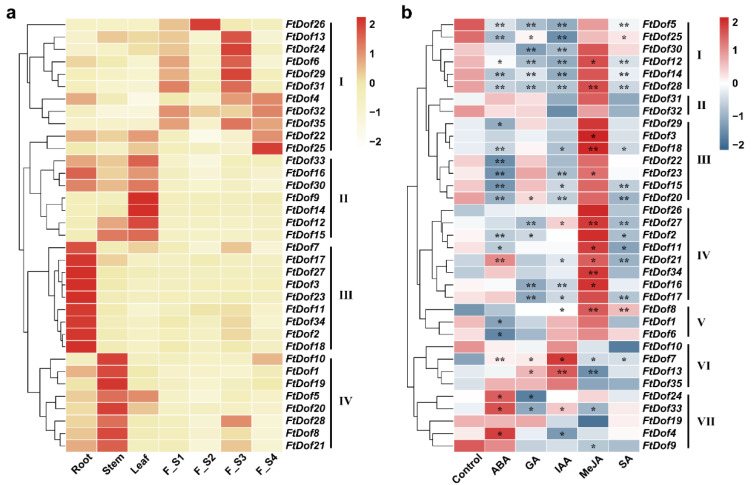
Heatmap of tissue specificity of Tartary buckwheat *Dof* genes and expression pattern after stimulation with exogenous hormones. (**a**) Expression pattern of *FtDofs* in Tartary buckwheat roots, stems, leaves and four developmental stages of fruit (F_S1, F_S2, F_S3 and F_S4). With the increase in gene expression, the colour of the bar shifts from white to red, as shown in the bar on the right. (**b**) Expression pattern of *FtDofs* after stimulation with exogenous hormones. The asterisk on the heatmap indicates a significant difference between the treatment and the control (Student’s *t*-test). * and ** represent 0.05 and 0.01 significance levels, respectively. With the increase in gene expression, the colour of the bar shifts from blue to red, as shown in the bar on the right.

The expression levels of 35 *FtDof* genes in Tartary buckwheat roots, stems, leaves and four developmental stages of fruit (F_S1, F_S2, F_S3 and F_S4) were characterised by qRT-PCR assay to gain insight into the potential roles of these genes (Figure 6a). Generally, the expression pattern of each gene varied greatly in different tissues, and they could be divided into four groups. Eleven genes in group I had relatively high expression levels in Tartary buckwheat fruit, but their dynamic expression patterns differed among the four developmental stages. The expression of *FtDof26* first increased and then decreased with fruit development, reaching the highest expression in the S2 stage. *FtDof13/24/6/29/31* were highly expressed in S1 and S3 stages, especially in the S3 stage. Additionally, the expression of *FtDof4/32/35/22/25* first decreased and then increased with fruit development. Seven genes in group II were all lowly expressed in fruits. Amongst them, *FtDof33/16/30* were highly expressed in roots, stems and leaves. The expression levels of *FtDof12* and *FtDof15* were higher in stems and leaves. *FtDof9* and *FtDof14* were specifically highly expressed in leaves. A notable detail was that all genes in group III were specifically expressed in roots and lowly expressed in other tissues. Eight genes in group IV had relatively high expression levels in stems, and they were expressed in other tissues to varying degrees.

### 3.7. Expression Pattern of FtDof Genes under Different Hormone Treatments

Analysis of the *cis*-acting elements in the promoter region of *FtDof* genes demonstrated that most genes contained abundant hormone-responsive elements. Thus, Tartary buckwheat seedlings were treated with five hormones (ABA, GA, IAA, MeJA and SA) to observe the response of *FtDof* genes. The statistical results showed that the expression of more than 40% genes changed significantly, specifically, 57.14% (5 upregulated and 15 downregulated) under ABA treatment, 42.86% (2 upregulated and 13 downregulated) under GA treatment, 54.29% (6 upregulated and 13 downregulated) under IAA treatment, 42.86% (12 upregulated and 3 downregulated) under MeJA treatment and 42.86% (2 upregulated and 13 downregulated) under SA treatment (Figure 6b).

*FtDof* genes were divided into seven groups (groups I–VII) in accordance with the overall expression patterns under hormone treatment. Group I contained six genes. Almost all of their expression levels changed under the five hormone treatments. The expression levels were downregulated under treatments with ABA, GA, IAA and SA. Except for *FtDof5* and *FtDof25*, the expression levels of other genes were all upregulated under MeJA treatment, especially those of *FtDof12* (*p* < 0.05) and *FtDof28* (*p* < 0.01). The expression levels of *FtDof31* and *FtDof32* in group II did not appear to be sensitive to any of the five hormone treatments. Group III was downregulated by ABA, IAA and SA treatments and upregulated by MeJA treatment. In group IV, the gene expression was upregulated under MeJA treatment and downregulated by SA treatment. Amongst the three genes in group V, the expression of *FtDof8* was significantly upregulated under IAA, MeJA and SA treatments, whilst the expression levels of *FtDof1* and *FtDof6* were significantly downregulated under ABA treatment. Groups VI and VII showed different patterns from those of groups I–V. The MeJA treatment was associated with the upregulation of *FtDof* genes in groups I–V and the downregulation of gene groups VI and VII. Conversely, the ABA and IAA treatments were associated with the overall downregulation of *FtDof* genes in groups I–V and an upregulation of genes in groups VI and VII. Overall, the 35 *FtDof* genes were generally downregulated by SA treatment.

### 3.8. GO and KEGG Enrichment Analysis of FtDof Proteins

**Figure 7:**
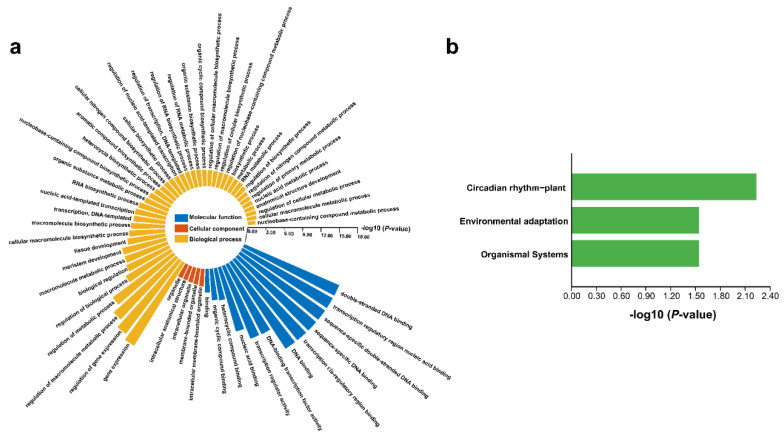
Enrichment analysis of *Ft*Dofs. (**a**) Gene ontology (GO) enrichment analysis of *Ft*Dofs. Blue, red and yellow represent three ontologies of GO, namely, molecular function, cellular component and biological process, respectively. (**b**) Kyoto Encyclopedia of Genes and Genomes (KEGG) enrichment analysis of *Ft*Dofs.

GO and KEGG enrichment analyses were carried out on the basis of the annotation database to investigate the functional category distribution of *Ft*Dof proteins (Table S6). The result of GO enrichment showed (Figure 7a) that *Ft*Dof proteins were distributed in three GO ontologies, namely, molecular function, cellular component and biological process. For molecular function, all but one transcription regulator activity belonged to the ‘binding’ category. The molecular functions were all related to the intracellular organelle. For biological processes, almost all items referred to ‘metabolic’, ‘biosynthetic’ or tissue development. The KEGG enrichment results showed that only one protein, *Ft*Dof14 (*FtPinG0009592200.01.T01*), was enriched and distributed in three pathways, namely, circadian rhythm-plant, environmental adaptation and organismal systems (Figure 7b and Table S6).

## 4. Discussion

### 4.1. Molecular Characterisation and Evolution of FtDof Proteinsin Tartary Buckwheat

Due to the special role of TFs in regulating gene expression, identifying and functionally characterising gene families that are widely present in the genome are important to further understand the growth and development of plants and their responses to environmental stimuli [44]. In the present study, 35 *Ft*Dofs were identified in the Tartary buckwheat genome. The number of *Ft*Dofs was similar to that identified for *A. thaliana* (36 *At*Dofs) [8], rice (30 *Os*Dofs) [8] and tomato (34 *Sl*Dofs) [12]. However, the genome sizes of these plants are quite different (Tartary buckwheat, 489.3 Mb [36]; *A. thaliana*, 125 Mb [45]; rice, 466 Mb [46]; and tomato, 900 Mb [47]), implying that the number of *Dof* proteinsis independent of genome size.

Phylogenetic analysis indicated that *Ft*Dofs could be classified into nine subgroups by using the optimal model of the ML method (Figure 2), consistent with the analysis of the *Dof* gene family in watermelon [48]. The exon–intron structure analysis revealed that the gene structures amongst different subgroups were substantially different, but generally, similar structures were observed within the subgroups (Figure 3a). Additionally, the number of introns in *FtDofs* was relatively small, ranging from 0 to 2, and most genes had no introns or only one intron. As mentioned in previous studies, the low number of introns may be related to the stress response [49]. As with the results of the exon–intron structure analysis, the arrangement of the *Ft*Dof motifs within the subgroup was generally consistent, but notable differences were found amongst the subgroups (Figure 3b). The findings indicate the possibility of great differences in the function of *Ft*Dofs amongst different subgroups. Nevertheless, all identified proteinshad a common motif (motif 1), the conserved *Dof* domain, which may be involved in binding to a particular promoter sequence [13]. Motif 10 is thought to be another conserved domain, which is likely to be a molecular hinge connecting the two domains as a serine stretch [4]. Moreover, almost every subgroup had its own unique and nearly conservative motifs. For example, motifs 3/4/5 only existed in the D1 subgroup, and motif 6 only existed in the C2.2 subgroup. Interestingly, in the C1 subgroup, except for *Ft*Dof29, all other proteinshad three motifs, motifs 1/8/10, which may lead to functional differences. Therefore, the existence of these motifs endows *Ft*Dofs with different functions, resulting in the functional differentiation of *Ft*Dofs [48].

Gene duplication is a common phenomenon in the evolution of angiosperms, usually including tandem and segmental duplication, which could lead to the expansion of the gene family [50]. Studies have revealed that tandem and segmental duplication events exist simultaneously in *Ptr*Dofs [51]. However, in *Ta*Dofs, tandem duplication events only existed at the ends of chromosomes [13]. In the present study, no tandem duplication event was observed in *Ft*Dofs, but 10 pairs of segmental duplication events were detected (Figure 4), similar to the results obtained for watermelon [48] and cotton [52]. This finding indicated that the expansion of *Ft*Dofs was mainly due to segmental duplication. Collinearity analysis can provide insights into the evolutionary history of species [41]. In the present study, the number of collinear gene pairs between the *Dof* proteinsof Tartary buckwheat and dicotyledons was much higher than that of monocotyledons, especially within soybean (Figure 5), consistent with previous research results on *ZF-HD* [53], *SPL* [54] and *NAC* [55] gene families of Tartary buckwheat.

### 4.2. Tissue-Specific Expression Characterisation of FtDof Genes in Tartary Buckwheat

Previous studies revealed that *Dof* genes usually had tissue-specific expression patterns [13,24,56], and similar phenomena were found in *FtDof* genes (Figure 6a). In the C1 subgroup, *AT5G62940* (*Dof5.6/HCA2*) was involved in the regulation of interfascicular cambium formation and vascular tissue development [57], and it was highly expressed in the root meristem and elongation zones [58]. Similarly, *AT5G60200* (*TMO6*) was expressed in the root meristem and elongation zones. The other two genes, *AT3G45610* and *AT2G28510*, were proven to be expressed in all three developmental regions (meristem, elongation and differentiation zones) of roots. *AT3G45610* and *AT5G60200* were both pericycle-specific [58]. Similar expression patterns were also found in the C1 subgroup; that is, all members in this subgroup were highly expressed in roots except for *FtDof29*, which was low in roots but highly expressed in the F_S3 stage of fruit development. Interestingly, compared with the other four genes, *FtDof29* lacked motif 8 and only contained motif 1 and motif 10. Therefore, the difference in the expression preference between *FtDof29* and other genes in the same subgroup was the absence of motif 8. *FtDof2* and *FtDof3* shared the same expression pattern, which was attributed to segmental duplication, indicating possible functional redundancy [48,59,60,61].

Five *A. thaliana Dof* genes in the B1 subgroup were highly expressed in root, stem and leaf tissues, and they played important biological functions. *AT2G37590* played important roles in the early developmental stages of vascular tissues and in the development of procambial cells in leaf primordia, roots and embryos [15]. *AT5G02460* was involved in the development of *A. thaliana* leaves, and it affected leaf axial patterns by promoting the transcription of *Revoluta* [62]. *AT1G07640* (*OBP2*) was expressed in the vascular system of all *A. thaliana* organs, with high expression in roots and leaves and relatively weak expression in stems [63]. Moreover, *AT3G55370* (*OBP3*) [64] and *AT2G28810* [58] were highly expressed in *A. thaliana* roots. Similar expression patterns were found in the other four *FtDof* genes of Tartary buckwheat in the B1 subgroup. *FtDof7* and *FtDof17* were highly expressed in roots. *FtDof5* and *FtDof33* were also expressed in roots, stems and leaves. Their expression levels in fruits were the lowest.

The C3 subgroup contained six genes, of which four *A. thaliana Dof* genes (*AT4G21030*, *AT4G21040*, *AT4G21050* and *AT4G21080*) were proven to be related to the development of seeds or mainly expressed in siliques [8,65]. Interestingly, the other two *FtDof* genes, *FtDof26* and *FtDof35*, were only highly expressed in fruit, showing exactly the same pattern as *A. thaliana*. Thus, *FtDof26* and *FtDof35* may play key roles in the fruit development of Tartary buckwheat, but this needs further experimental verification.

### 4.3. Expression Pattern of FtDof Genes in Response to Hormones

The *cis*-acting elements present in the promoter region play pivotal roles in gene expression [66]. In the present study, several hormone-responsive elements were found in almost all promoter regions of *FtDof* genes, including ABA-, GA-, IAA-, MeJA- and SA-responsive elements (Table S4). The qRT-PCR analysis showed that the expression patterns of *FtDof* genes were inconsistent under different treatments. Specifically, *FtDof* genes were mainly upregulated under MeJA treatment but downregulated under other hormone treatments. Numerous previous studies have shown that various hormones usually regulate the expression of *Dof* genes. The transcription level of *OBP2* in *Arabidopsis* [63] and *VvDOF3* in grape [67] increased significantly upon treatment with MeJA. However, the expression of *BrDof2.4* in Chinese flowering cabbage was suppressed under MeJA treatment [68]. Similarly, the expression patterns of *Dof* genes in response to exogenous ABA [24], GA [69], IAA [13] and SA [70] treatments were complex and changeable in other plants. Additionally, studies have illustrated no direct correlation between the number of *cis*-acting elements and gene expression patterns [71,72]. In the present study, the expression levels of three genes, namely, *FtDof7*, *FtDof12* and *FtDof28*, changed significantly under treatment with five exogenous hormones. However, few hormone-responsive elements were present in their promoter regions. In particular, *FtDof7* had only three *cis*-elements, TCA (SA-responsive), W box (ABA-responsive) and ABRE (ABA-responsive), implying the presence of complex regulatory mechanisms affecting the expression of *FtDof* genes.

## 5. Conclusions

In this study, 35 *Ft*Dof proteins were identified on the basis of the Tartary buckwheat genome database, and these genes were classified into nine subgroups. The *FtDof* genes within each subgroup shared similar gene structure and motif arrangement, and they were distinguishable amongst subgroups. Some motifs were uniquely or even conservatively found in individual subgroups, presumably with specialised functions. Segmental duplication was the primary driving force for the evolution of the *Ft*Dof gene family. Moreover, the *FtDof* genes had clear expression specificity and preference in different tissues and fruit development stages, most of which were significantly regulated by the treatment with exogenous hormones. This finding indicates that *FtDof* genes may be involved in multiple biological processes during the growth and development of Tartary buckwheat. In conclusion, this study provides a foundation for further exploration of the functional characteristics of the Tartary buckwheat *Dof* gene family and its role in growth and development and resisting stress. It will also be helpful in improving varieties of Tartary buckwheat in the future.

## Figures and Tables

**Table 1:** *Dof* family genes in Tartary buckwheat.

Gene Name	Gene ID	Chr Location	CDS Length (bp)	Protein Length (aa)	Mw (kDa)	*p*I	Subcellular Location
*FtDof1*	FtPinG0002490200.01.T01	Ft1:6984896-6985480	555	184	20.91976	9.23	Nuclear
*FtDof2*	FtPinG0005819100.01.T01	Ft1:11995949-11997181	1023	340	37.16502	6.88	Nuclear
*FtDof3*	FtPinG0006052400.01.T01	Ft1:26283542-26284756	1029	342	37.13006	7.13	Nuclear
*FtDof4*	FtPinG0000038100.01.T01	Ft1:26894633-26895469	786	261	26.95576	6.29	Nuclear
*FtDof5*	FtPinG0006014000.01.T01	Ft1:35519701-35521178	1116	371	39.08230	9.15	Nuclear
*FtDof6*	FtPinG0000383700.01.T01	Ft1:51008719-51009996	828	275	30.39486	8.88	Nuclear
*FtDof7*	FtPinG0007854200.01.T01	Ft2:12819999-12820866	735	244	26.69874	9.84	Nuclear
*FtDof8*	FtPinG0009668700.01.T01	Ft2:46641998-46642846	846	281	31.58071	4.46	Nuclear
*FtDof9*	FtPinG0003838400.01.T01	Ft2:56803328-56804871	729	242	26.43628	9.23	Nuclear
*FtDof10*	FtPinG0000870700.01.T01	Ft2:60683203-60684009	792	263	29.30853	7.13	Nuclear
*FtDof11*	FtPinG0003987600.01.T01	Ft3:4327917-4328955	879	292	32.09061	8.60	Nuclear
*FtDof12*	FtPinG0006042300.01.T01	Ft3:8679735-8681642	1089	362	39.37413	6.55	Nuclear
*FtDof13*	FtPinG0001985100.01.T01	Ft3:17940022-17941017	954	317	34.10505	8.84	Nuclear
*FtDof14*	FtPinG0009592200.01.T01	Ft3:19407541-19409658	846	281	31.00104	9.56	Nuclear
*FtDof15*	FtPinG0006943800.01.T01	Ft3:20185865-20187740	1209	402	44.58969	7.58	Nuclear
*FtDof16*	FtPinG0001746600.01.T01	Ft3:38071212-38072846	969	322	35.98977	8.57	Nuclear
*FtDof17*	FtPinG0006517000.01.T01	Ft3:45271193-45272090	756	251	27.20933	9.32	Nuclear
*FtDof18*	FtPinG0001221400.01.T01	Ft3:52038929-52039519	519	172	19.48991	9.08	Nuclear
*FtDof19*	FtPinG0002126900.01.T01	Ft3:54786097-54786807	696	231	26.33179	4.83	Nuclear
*FtDof20*	FtPinG0006352100.01.T01	Ft4:3820883-3823102	1305	434	47.49093	6.05	Nuclear
*FtDof21*	FtPinG0000044800.01.T01	Ft4:5503745-5504860	1029	342	37.27505	6.00	Nuclear
*FtDof22*	FtPinG0005157600.01.T01	Ft4:15029533-15030375	750	249	27.95802	9.15	Nuclear
*FtDof23*	FtPinG0000644200.01.T01	Ft5:3393885-3394904	831	276	31.16242	6.13	Nuclear
*FtDof24*	FtPinG0007868100.01.T01	Ft5:39770127-39770799	639	212	23.72748	8.61	Nuclear
*FtDof25*	FtPinG0007057700.01.T01	Ft6:7340538-7343583	1416	471	50.79071	6.20	Nuclear
*FtDof26*	FtPinG0006338000.01.T01	Ft6:10848783-10849422	423	140	15.48469	9.21	Nuclear
*FtDof27*	FtPinG0000330300.01.T01	Ft6:49780770-49782567	1008	335	36.87670	9.15	Nuclear
*FtDof28*	FtPinG0002571600.01.T01	Ft7:33993961-33994407	393	130	14.84745	9.32	Nuclear
*FtDof29*	FtPinG0009543500.01.T01	Ft7:50345602-50346563	858	285	31.47978	8.16	Nuclear
*FtDof30*	FtPinG0002293400.01.T01	Ft8:1391182-1394031	990	329	36.68763	7.62	Nuclear
*FtDof31*	FtPinG0002167800.01.T01	Ft8:3967827-3968546	720	239	26.66127	5.13	Nuclear
*FtDof32*	FtPinG0008252900.01.T01	Ft8:15269916-15270644	675	224	23.02970	8.57	Nuclear
*FtDof33*	FtPinG0004209600.01.T01	Ft8:18068180-18069652	1092	363	37.55589	9.03	Nuclear
*FtDof34*	FtPinG0005937800.01.T01	Ft8:47262582-47263547	702	233	26.2462	6.44	Nuclear
*FtDof35*	FtPinG0000702500.01.T01	Ft8:47316870-47317455	531	176	19.33684	9.39	Nuclear

Chr, chromosome; CDS, coding sequence; bp, base pair; aa, amino acid; Mw, molecular weight; *p*I, isoelectric point.

## Data Availability

The data presented in this study are available in the article and its supplementary materials.

## Supplementary Materials

The following supporting information can be downloaded at: https://www.mdpi.com/article/10.3390/biology11020173/s1, Table S1: The primers of *FtDofs* used for qRT-PCR; Table S2: The CDS and protein sequences of *FtDofs* identified in this study; Table S3: The putative motifs identified in *Ft*Dof proteins by MEME; Table S4: The *cis*-acting elements in the promoter regions of *FtDofs*; Table S5: Orthologous gene pairs between Tartary buckwheat and seven other species; Table S6: Gene Ontology (GO) and Kyoto Encyclopedia of Genes and Genomes (KEGG) enrichment analysis of *Ft*Dof proteins.
